# Successful Surgical Repair With Frozen Elephant Trunk for Acute Type A Aortic Dissection After Percutaneous Closure of a Patent Ductus Arteriosus

**DOI:** 10.7759/cureus.87293

**Published:** 2025-07-04

**Authors:** Hironaga Shiraki, Soichiro Henmi, Takeshi Inoue, Kenji Okada

**Affiliations:** 1 Cardiovascular Surgery, Kobe University, Kobe, JPN; 2 Cardiovascular Surgery, Hyogo Prefectural Awaji Medical Center, Sumoto, JPN

**Keywords:** amplatzer plug device, frozen elephant trunk technique, pda closure, positive arterial remodeling, type a aortic dissection

## Abstract

Acute type A aortic dissection (ATAAD) following percutaneous closure of a patent ductus arteriosus (PDA) is an exceedingly rare complication, with no documented successful treatment outcomes in the existing literature. We report a successful case of surgical repair for ATAAD in a 74-year-old male patient who had previously undergone percutaneous PDA closure. Utilizing a translocated total arch replacement with frozen elephant trunk (FET) technique, we achieved chronic aortic remodeling without the need to remove the PDA closure device.

## Introduction

Transcatheter closure of patent ductus arteriosus (PDA) has become the standard treatment even in adult cases [1]. However, this procedure is associated with various complications, including device embolization, malposition, partial obstruction of the pulmonary artery, hemolysis, and infective endocarditis [2,3]. Among these, acute type A aortic dissection (ATAAD) is an exceedingly rare but life-threatening event [4]. In one report, Baker et al. described a case of a 66-year-old woman who developed fatal ATAAD 12 years after transcatheter PDA closure, with postmortem findings revealing a large intimal tear adjacent to the PDA occluder [5]. This case represents one of the first documented instances of late-onset ATAAD following PDA device closure. The development of ATAAD may be related to factors such as device mismatch and vascular fragility, necessitating prompt recognition and management.

Here, we present a case of a patient who developed ATAAD following transcatheter PDA closure but was successfully treated with surgical repair and exhibited favorable long-term downstream aortic remodeling. This case highlights the risk factors for aortic dissection in PDA closure, the importance of early diagnosis, and optimal management strategies.

## Case presentation

**Figure 1:**
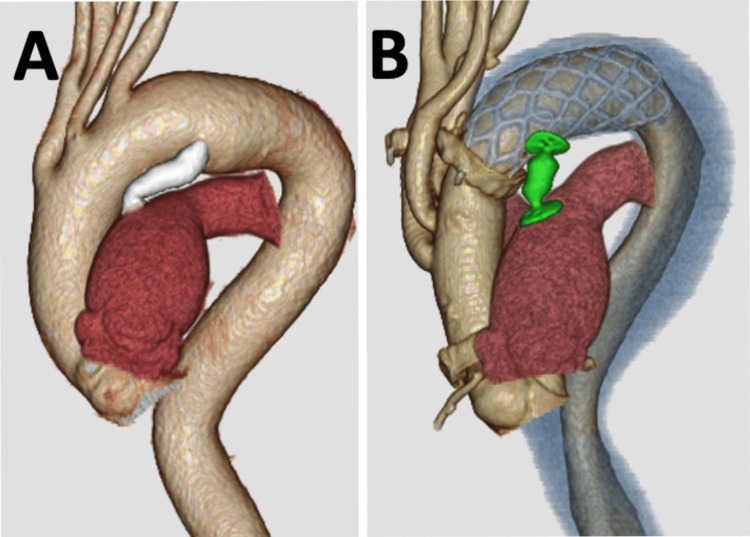
3D computed tomography scan Before the procedure, a large PDA was shown by computed tomography scan (A). Postoperative computed tomography scan revealed successful coverage of the intimal tear and thrombosis in the false lumen (B). (Green device: AVPII; Colored blue lumen: Thrombosis in false lumen) PDA: Patent ductus arteriosus; AVPII: Amplatzer Vascular Plug II

A 74-year-old man presented with progressive breathlessness, diagnosed as congestive heart failure. Computed tomography (CT) imaging revealed a large and long PDA (Figure 1A).

**Figure 2:**
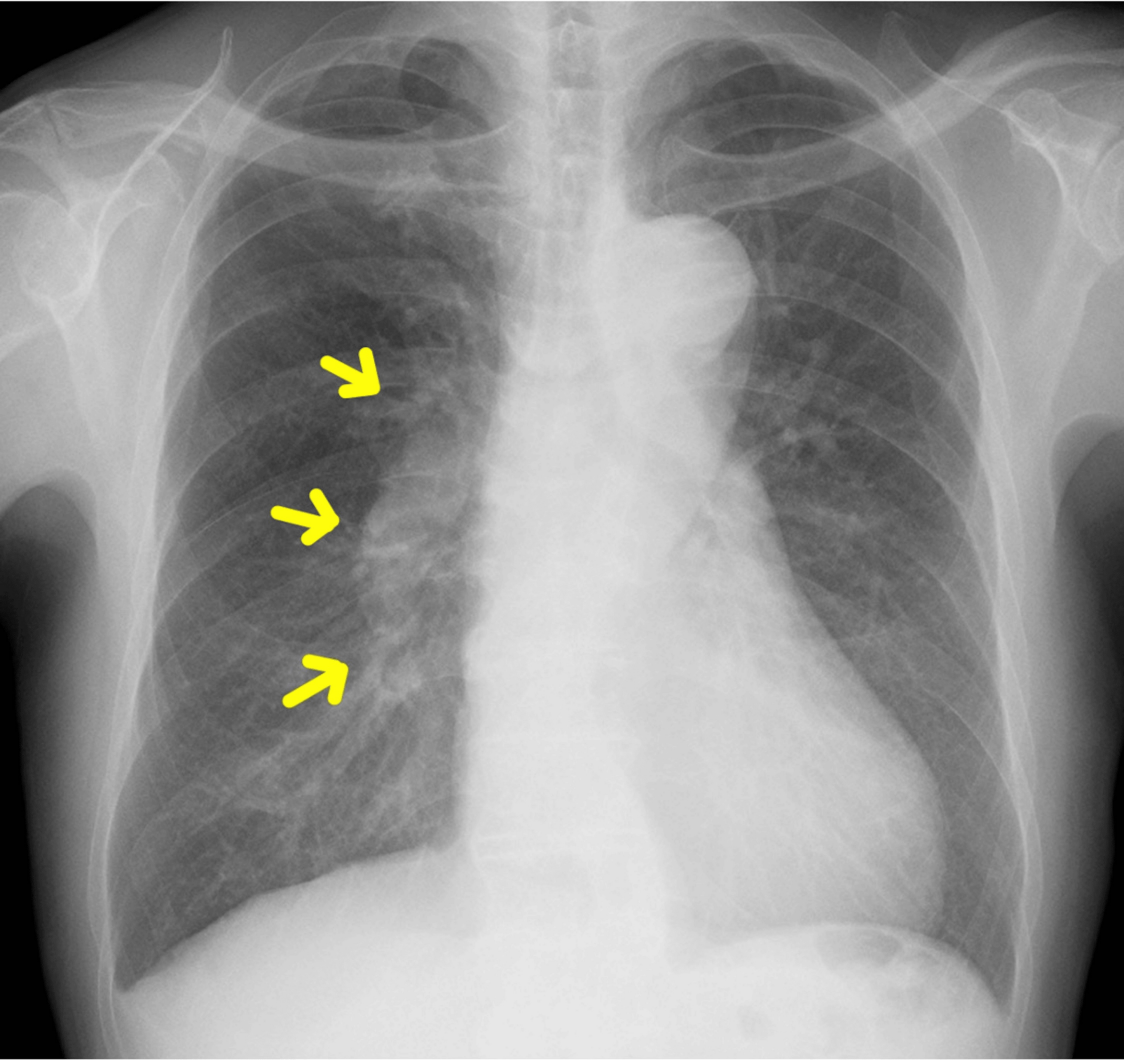
Preoperative chest X-ray The yellow arrows indicate mildly increased pulmonary vascular markings, consistent with pulmonary overcirculation due to a left-to-right shunt from the PDA. No signs of pulmonary congestion or advanced pulmonary vascular disease were observed. PDA: Patent ductus arteriosus

Preoperative evaluation revealed no significant valvular abnormalities on cardiac 2D echocardiography. Right heart catheterization demonstrated a pulmonary-to-systemic blood flow ratio (Qp/Qs) of 2.12 and a pulmonary vascular resistance (PVR) of 182 dynes/sec/cm^-3^, indicating a significant left-to-right shunt without evidence of irreversible pulmonary hypertension. The preoperative chest X-ray showed mildly increased pulmonary vascular markings, suggestive of pulmonary overcirculation, but no findings indicative of advanced pulmonary vascular disease (Figure 2).

**Figure 3:**
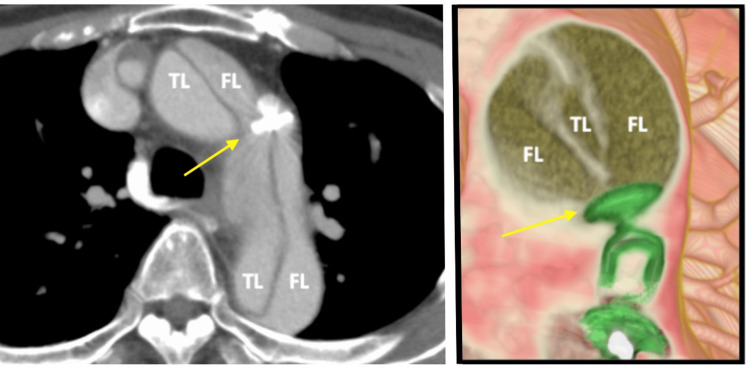
Intimal tear The following day after PDA closure, computed tomography scan revealed acute type A aortic dissection with intimal tear at the site of AVPII (yellow arrows). The device was migrated into the false lumen of the aorta. (Green device: AVPII) TL: True lumen; FL: False lumen; PDA: Patent ductus arteriosus; AVPII: Amplatzer Vascular Plug II

Transvenous percutaneous closure of the PDA was performed using a 16-mm Amplatzer Vascular Plug II (AVPII) (St. Jude Medical, St. Paul, USA), with a final aortogram confirming successful closure. However, the day following the procedure, the patient complained of sudden, severe back pain. The emergent enhanced CT scan revealed ATAAD extending from the ascending aorta to the iliac arteries, with an aortic tear at the site of the AVPII in the distal aortic arch, where the device had migrated into the false lumen (Figure 3). Urgent surgical intervention was performed.

Via median sternotomy, cardiopulmonary bypass was established through the ascending aorta and right atrium. Under moderate hypothermia (rectal temperature at 29°C) with circulatory arrest [6,7], the aorta was opened and three supra-aortic vessels were cannulated for selective cerebral perfusion. An intimal tear was identified in the small curvature of the distal aortic arch, coinciding with the AVPII deployment site. The aortic arch was transversely dissected between the left common carotid artery (LCCA) and left subclavian artery (LSCA). The LSCA was resected at its origin to facilitate the insertion of a 29-mm FROZENIX (Japan Lifeline Co., Ltd., Tokyo, Japan) of 90 mm length into the true lumen of the descending thoracic aorta from zone 2 to cover the intimal tear. After deployment of the frozen elephant trunk (FET), the distal aortic stump was anastomosed to a 24-mm, three-branch Dacron graft (Japan Lifeline Co., Ltd., Tokyo, Japan) using Teflon felt and 4-0 polypropylene running suture. The proximal aorta was transected 1 cm distal to the sinotubular junction, with re-approximation performed using Teflon felt and BioGlue (CryoLife Inc., Kennesaw, USA) on the adventitia and 4-0 polypropylene running suture within the false lumen. Following completion of the proximal anastomosis and the return of spontaneous cardiac activity, in situ reconstruction of the LSCA, LCCA, and innominate artery was performed.

**Figure 4:**
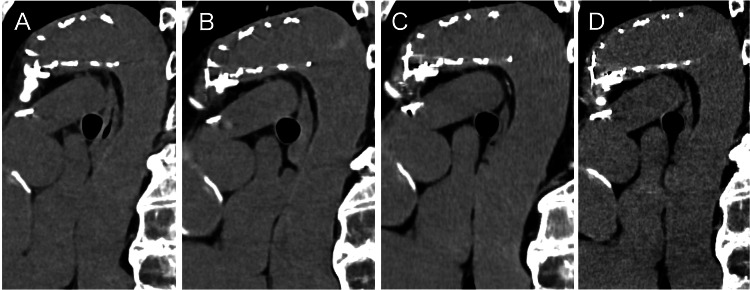
Downstream aorta after operation The computed tomography scan of the downstream aorta at (A) six months after operation, (B) two years after operation, (C) four years after operation, and (D) six years after operation.

The patient's postoperative course was uneventful, with an enhanced CT scan before discharge demonstrating successful coverage of the intimal tear and thrombosis in the false lumen (Figure 1B). Six years post-surgery, the patient remains well, without any aortic events, and follow-up CT imaging indicated stable downstream aortic dimensions and favorable remodeling (Figure 4). At six months postoperatively, the diameters of the distal arch and the descending aorta at the level of the aortic valve measured 35.3 mm and 33.0 mm, respectively. At six years following surgery, these measurements were 34.7 mm and 30.2 mm, respectively, indicating stable aortic remodeling over time.

## Discussion

This report documents the successful surgical repair of ATAAD after percutaneous PDA closure. Previously, cardiologists at our institution reported on this case [8], primarily focusing on imaging findings with limited discussion on the surgical procedure. Herein, we provide a comprehensive discussion, emphasizing the surgical technique employed and the long-term aortic remodeling outcomes following the procedure.

In principle, complete exclusion of the entry tear is paramount during surgical repair for ATAAD. In this case, achieving complete tear exclusion would have been feasible if the distal aortic stump had been created at a level distal to the PDA. However, we encountered challenges due to the necessity of anastomosing in the deep and severely fragile distal aortic arch surrounding the PDA tissue, in addition to the need for additional procedures to remove the AVPII and close the calcified foramen of the PDA on the pulmonary artery. These complex surgical maneuvers were perceived as particularly challenging, especially in the context of a life-saving procedure.

To our knowledge, there have been no previous reports describing the placement of an FET directly over a previously implanted PDA closure plug, as performed in this case. In contrast, a few reports have described the reverse sequence, where thoracic endovascular aortic repair (TEVAR) was initially performed for type B aortic dissection associated with PDA, and persistent endoleak originating from the PDA was subsequently managed successfully by percutaneous plug closure, resulting in resolution of the endoleak and favorable distal aortic remodeling [9]. These prior experiences support the concept that appropriate closure of PDA-related flow may contribute to improved aortic remodeling in selected patients.

Additionally, the selection of an appropriate size for the FET is also deemed crucial. Furthermore, in this case, we were able to ascertain the diameter of the descending aorta from a previous CT image obtained before percutaneous PDA closure (Figure 1A). This information facilitated the selection of the optimal size for the FET, potentially contributing to improved outcomes and aortic remodeling. However, in certain emergency settings of acute aortic dissection, detailed preoperative measurements for optimal FET sizing and distal positioning may not always be possible. As a result, there is a theoretical risk of distal oversizing or unintended distal landing positions. If progressive dilatation of the distal aorta or distal stent graft induced new entry (d-SINE) develops postoperatively, complementary TEVAR after FET, as described by Mylonas et al., may serve as a valuable secondary intervention to promote distal aortic remodeling [10]. Therefore, continuous surveillance remains essential even after FET deployment.

Several factors could have contributed to the occurrence of acute aortic dissection following percutaneous PDA closure. Firstly, damage to the aortic wall may have occurred due to the placement of the device on the aortic side and excessive traction of the device towards the pulmonary artery side, particularly given the length of the PDA. Secondly, degenerative aortopathy could have predisposed the patient to aortic dissection. The CT imaging revealed dilation of the aortic arch to 42 mm and arteriosclerosis in the aorta. Thirdly, the patient had a long history of hypertension. Achieving strict blood pressure control post-device deployment can be challenging due to the abrupt hemodynamic changes following PDA closure.

Percutaneous closure is generally considered difficult in cases with short, wide, deformed, and calcified PDAs. Furthermore, the presence of associated aneurysmal formation or infective endocarditis is generally regarded as an indication for surgical repair [11]. Open surgery via thoracotomy would require cardiopulmonary bypass and likely circulatory arrest, and it is often associated with significant invasiveness and perioperative risks [12]. Patients with preoperative heart failure, pulmonary hypertension, or low ejection fraction are at increased risk for developing postoperative low-output syndrome or congestive heart failure. Therefore, the minimally invasive nature of TEVAR, particularly its avoidance of cardiopulmonary bypass, may be associated with enhanced postoperative recovery compared to conventional open repair. Several case reports have described PDA closure performed using TEVAR [12-14]. The absence of catheter manipulation across the PDA in TEVAR has led to suggestions that it may be performed more safely than percutaneous device closure in selected cases [15].

However, in cases with concomitant cardiac disease requiring surgical intervention or in those with infective endocarditis, open surgery remains the preferred approach. Moreover, the structural fragility of the aortic wall around the PDA may predispose patients to complications such as retrograde aortic dissection or d-SINE after TEVAR. The indications for TEVAR specifically for PDA closure have not yet been clearly established, and long-term outcomes remain uncertain. Thus, careful consideration is required when selecting the appropriate treatment. 

In this case, although a zone 2 landing is needed due to the short distance between the left subclavian artery and PDA, it is anticipated that PDA exclusion using TEVAR can be achieved with relative ease, considering anatomical factors.

## Conclusions

The successful entry closure with FET over the vascular plug resulted in favorable long-term aortic remodeling, demonstrating the feasibility of this approach in complex cases. Several risk factors, including device-related mechanical stress, pre-existing aortopathy, and hypertension, may contribute to aortic dissection in similar scenarios, necessitating a cautious patient selection process. Given the potential risks associated with percutaneous PDA closure, alternative approaches such as TEVAR should be considered, particularly in adults with large, calcified, or arteriosclerotic PDA. Further clinical experience and research are necessary to optimize treatment strategies for high-risk PDA cases and improve long-term patient outcomes.
